# Nationwide Trends in Hospitalizations for Sudden Cardiac Arrest Before and During the COVID Outbreak

**DOI:** 10.3390/jcm14217517

**Published:** 2025-10-23

**Authors:** Sarah Daoudi, Ariel Furer, Kevin John, Fadi Chalhoub, Jennifer Chee, Margaret Infeld, Gabby Elbaz-Greener, Munther Homoud, James Udelson, Christopher Madias, Guy Rozen

**Affiliations:** 1 Division of Cardiology, Tufts Medical Center, Boston, MA 02111, USA; sarah.daoudi@einsteinmed.edu (S.D.);; 2 Baystate Medical Center, Springfield, MA 01199, USA; 3 Department of Cardiology, Hadassah Medical Center, Jerusalem 91120, Israel

**Keywords:** sudden cardiac arrest, ventricular fibrillation, ventricular tachycardia, in-hospital mortality, out-of-hospital cardiac arrest, SARS-CoV-2, epidemiology

## Abstract

**Background/Objectives**: Sudden cardiac arrest (SCA) accounts for ~50% of cardiovascular mortality in the U.S. Cardiovascular complications are common in acute and post-acute COVID-19 infection. We aimed to examine nationwide trends in SCA-related hospitalizations in the United States before and during the COVID-19 outbreak. **Methods**: Using data from the National Inpatient Sample, we conducted a retrospective analysis of hospitalizations for SCA in the U.S. between 2016 and 2020. Sociodemographic and clinical characteristics and in-hospital mortality were compared between the pre-COVID (2016–2019) and COVID (2020) eras. Multivariable analysis was performed to identify factors associated with mortality. **Results**: Among a weighted total of 153,100 SCA hospitalizations between 2016 and 2020, the median age was 65 years, 62.7% were male, and 66.6% were white. There was a trend towards fewer hospitalizations in 2020 compared to prior years (*n* = 28,585 vs. *n*_average_ = 32,129, *p* = 0.07). In-hospital mortality remained unchanged between the pre-COVID and COVID eras (47.7% vs. 47.3%, *p* = 0.66). Increased mortality was associated with female sex (OR: 1.21; 95% CI: 1.15–1.28; *p* < 0.001), non-white race (OR: 1.24; 95% CI: 1.15–1.28; *p* < 0.001), history of renal failure (OR: 1.08; 95% CI: 1.02–1.15; *p* = 0.007), and diabetes (OR: 1.32; 95% CI: 1.25–1.39; *p* < 0.001). In 2020, 1.5% of the study population was diagnosed with COVID-19 infection, which was found to be independently associated with increased in-hospital mortality (OR: 1.57; 95% CI: 1.27–1.95; *p* < 0.001). **Conclusions**: In 2020, there was a trend towards a decrease in hospitalizations for SCA, while COVID-19 infection was independently associated with higher in-hospital mortality among patients admitted with SCA.

## 1. Introduction

Sudden Cardiac Arrest (SCA) is a leading cause of death in the United States, with annual incidence of SCA approximating 600,000 cases per year, responsible for approximately 13% of all deaths in the United States [1]. Common etiologies for SCA include coronary heart disease, myocarditis, inherited cardiomyopathies and cardiac vascular anomalies [2]. In early 2020, the CDC first published information about the novel coronavirus (2019-nCoV) outbreak caused by the SARS-CoV-2 virus [3]. In the months and years that followed, it has been shown that cardiovascular complications are a common manifestation of acute and post-acute COVID-19 infection due to the virus’s tendency to target the cardiovascular system, among other body systems [4].

A small study, analyzing the first weeks of the COVID-19 pandemic data, documented increased rates of SCA during the beginning of the pandemic, with evidence suggesting worse outcomes and elevated mortality in affected patients [5]. In this study, communities with high COVID-19 mortality demonstrated an increased incidence of out-of-hospital cardiac arrest (OHCA) [5]. Additionally, review studies comparing OHCA before and during the COVID-19 pandemic have found increased arrests at home, decreased survival and neurological outcomes, reduced AED use and resuscitation rates, and overall worse patient prognosis both out-of-hospital and in-hospital [6,7]. While this trend was observed in the general population, studies indicated no significant increase in SCA rates among individuals under 40 years of age following the pandemic’s onset [8]. Among hospitalized COVID-19 patients, SCA and ventricular arrhythmias were associated with substantial morbidity and mortality [9]. However, these reports should be interpreted with caution due to their limited scope and relatively small sample sizes.

The aim of this study is to investigate nationwide trends in SCA-related hospitalizations across the United States, comparing patient characteristics and outcomes, including in-hospital mortality, between pre-pandemic and COVID-19 era periods.

## 2. Methods

### 2.1. Data Sources

The data were drawn from the Nationwide Inpatient Sample (NIS), a database developed for the Healthcare Cost and Utilization Project (HCUP) sponsored by the Agency for Healthcare Research and Quality (AHRQ, Rockville, MD, USA) [10]. The NIS is the largest collection of all-payer data on inpatient hospitalizations in the United States. The dataset includes an approximate 20% stratified sample of all inpatient discharges from U.S. hospitals, representing the entire population of patients, hospitalized in the US. The database provides de-identified information for each hospital stay. This information includes patient-level and hospital-level factors: patients’ demographic characteristics; primary and secondary diagnoses and procedures; comorbidities; length of stay (LOS); hospital region; hospital teaching status; hospital bed size; and cost of hospitalization. Accurate national estimates are calculated based on this sample, using the patient-level and hospital-level sampling weights that are provided by the NIS. We obtained data for the years 2016 to 2020 (full calendar year).

### 2.2. Study Population

**Figure 1:**
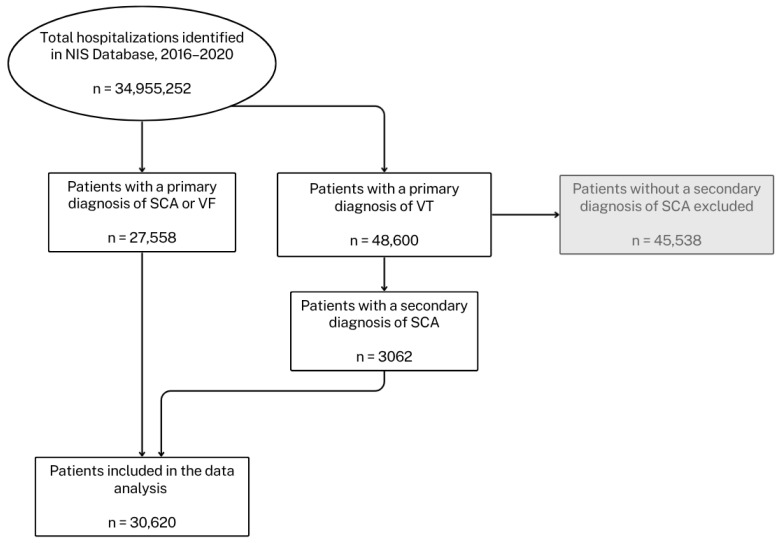
Flowchart showing selection of hospitalizations for sudden cardiac arrest (SCA) from the National Inpatient Sample (NIS), 2016–2020. Patients were identified using ICD-10 diagnosis codes. Patients with missing age, sex, or mortality status (~0.03% of SCA patients identified in the NIS database) were excluded from the dataset. Final sample size: 30,620. Arrows indicate direction of cohort refinement. NIS = National Inpatient Sample; SCA = sudden cardiac arrest; VF = ventricular fibrillation; VT = ventricular tachycardia.

The NIS Database reports diagnoses and procedures through the International Classification of Diseases-10th Revision-Clinical Modification and Procedure Coding system (ICD-10-CM/PCS). For each index hospitalization, the database provides a principal discharge diagnosis and a maximum of 39 additional diagnoses, in addition to a maximum of 25 procedures [10]. We selected patients 18 years of age or older hospitalized in the U.S. between 2016 and 2020 with a primary diagnosis reflecting SCA. We identified patients with a primary diagnosis of SCA based on ICD-10-CM codes for SCA (I46, I46.2, I46.8, I46.9), VF (I49.0, I49.01, I49.02), and VT (I47.20, I47.21, I47.29). For patients with VT as the primary diagnosis, inclusion required a secondary diagnosis (diagnoses 2–29 of SCA (I46, I46.2, I46.8, I46.9)). Patients were excluded if age, sex, or mortality status was missing (~0.03% of identified cases). The final cohort was then divided into pre-COVID (2016–2019) and COVID (2020) periods. A detailed flow chart outlining the selection process and exclusions is provided in Figure 1.

### 2.3. Study Outcomes

Utilizing the ICD-10-CM diagnosis codes, we collected and analyzed baseline characteristics of patients with a primary diagnosis of SCA, including common comorbidities (hypertension, congestive heart failure, diabetes, renal failure, COVID-19, ischemic heart disease, acute coronary syndrome, peripheral vascular disease, presence of cardiac pacemaker, and presence of implantable cardiac defibrillator) to compare trends between the pre-COVID era (2016–2019) and the COVID era (2020) (See Appendix A, Table A1 for ICD-10 CM diagnosis and procedure codes). Patient demographic data including sex, age, race, and income percentile, as well as hospital-level data including hospital region, hospital teaching status, and hospital bed size, were also collected. To calculate the Deyo–Charlson comorbidity index (Deyo-CCI), an additional list of comorbidities was identified from the database by using ICD-10-CM codes (detailed information on Deyo-CCI provided in Appendix A, Table A2). Deyo-CCI is a modification of the Charlson comorbidity index. It contains 17 comorbid conditions with differential weights, with a total score ranging from 0 to 33. Higher Deyo-CCI scores indicate a greater burden of comorbid diseases and are associated with mortality, 1 year after admission [11,12]. The index has been used extensively in studies from administrative databases, with proven validity in predicting short- and long-term outcomes [13,14].

We also utilized the ICD-10-PCS procedure codes to identify in-hospital interventions and outcomes for these patients, including coronary catheterizations, pacemaker or ICD implantations, as well as length of hospital stay and in-hospital mortality, to compare trends between the pre-COVID and COVID eras. Multivariable analysis was performed to identify independent associations with in-hospital mortality.

### 2.4. Statistical Analysis

Discharge-level weight files (“DISCWT”) provided by AHRQ were used to reflect national estimates. The Chi-squared test and Wilcoxon Rank Sum test were used to compare categorical variables and continuous variables, respectively. To account for hospital-level clustering of discharges, we generated a two-level mixed-effects logistic regression model to identify factors independently associated with mortality. Congruent with HCUP NIS design, hospital identification number was employed as a random effect with patient-level factors clustered within hospital-level factors. Candidate variables included patient-level characteristics, Deyo–CCI, and hospital-level factors. A *p*-value of <0.05 was considered significant. All tests were two-tailed. Statistical analysis was conducted using SPSS software version 25.0 (IBM Corp., Armonk, NY, USA) and R version 4.3.2 (R Foundation for Statistical Computing, Vienna, Austria).

## 3. Results

### 3.1. Trends in SCA Hospitalizations 2016–2020

Out of the 34,955,252 U.S. hospitalizations documented in the NIS database (20% sample of all U.S. hospitalizations) between 2016 and 2020, a total of 30,620 hospitalizations with a primary discharge diagnosis of SCA were included in the study based on the inclusion criteria (Figure 1). After weighting, these represent an estimated total of 153,100 hospitalizations following SCA in the U.S. between 2016 and 2020. For these patients, the median age was 65 (IQR: 21) years, 63% were male, and 67% were white. The most common comorbidities were hypertension (68.4%), diabetes mellitus (33.8%), congestive heart failure (29.1%), and renal failure (28.7%). In 2020, 1.5% of patients in the study population were diagnosed with COVID-19 infection. Table 1 presents the demographic and clinical characteristics of patients compared between the two study periods (See Supplementary Table S1 for baseline characteristics for the years 2016–2020).

In 2020, the data show a trend towards decreased hospitalizations for SCA, compared to the pre-COVID era that did not reach statistical significance (*n* = 28,585 vs. *n*_average_ = 32,129, *p* = 0.07). Notably, the drop in hospitalizations in 2020 comes in contrast to the uptrend in SCA hospitalizations across the U.S. during the 4-year period from 2016 to 2019 (Figure 1). When comparing patient characteristics between the pre-COVID period (2016–2019) and 2020, there was a significant shift in age distribution (*p* = 0.007). The proportion of patients aged 18–44 increased slightly (10.7% vs. 11.4%), while those aged 75 and older decreased (27.2% vs. 25.2%). No significant differences were observed in gender distribution between the pre-COVID and COVID periods.

In addition, there was a statistically significant increase in the incidence of acute coronary syndrome (14.7% vs. 18.5%, *p* < 0.001) and congestive heart failure (28.8% vs. 30.3%; *p* = 0.03) and decrease in the incidence of prior ischemic heart disease (23.7% vs. 22.0%; *p* = 0.0091 and diabetes (34.1% vs. 32.6%; *p* = 0.03) among patients admitted following SCA in 2020 compared with the pre-COVID era (2016–2019). Finally, during the COVID outbreak year, more patients were treated in urban teaching hospitals and small hospitals compared to earlier era (*p* < 0.001 for both).

### 3.2. Outcomes of SCA Hospitalizations 2016–2020

**Figure 2:**
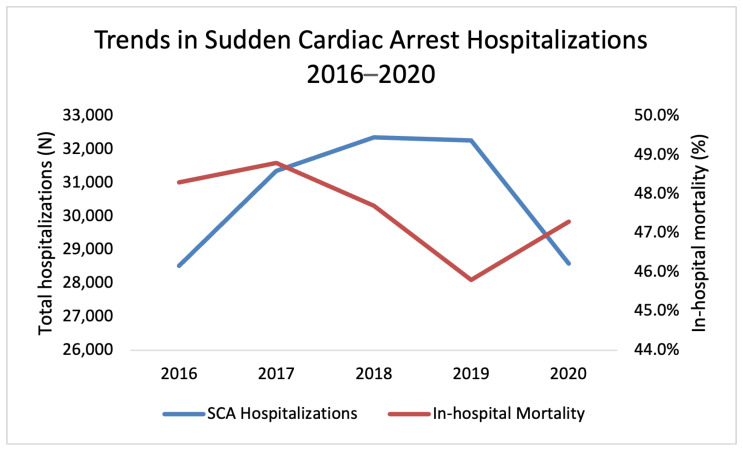
Annual sudden cardiac arrest (SCA) hospitalizations and in-hospital mortality in the U.S. from 2016 to 2020, based on data from the National Inpatient Sample (NIS). Hospitalizations increased from 2016 to 2019 but declined in 2020. In-hospital mortality decreased through 2019, then rose in 2020. SCA = sudden cardiac arrest.

As shown in Table 2, there was no difference in patients’ in-hospital mortality (47.7% vs. 47.3%, *p* = 0.66) between the pre-COVID and COVID era despite a gradual decrease in the mortality rate from 2016 to 2019, followed by an increase in the mortality rate in 2020 (Figure 2, See Supplementary Table S2). There was, however, a significant increase in the length of stay (*p* = 0.008) and total charges (<0.001) per hospitalization during the COVID year. In addition, there was an increase in the number of patients being transferred to Home Health Care after discharge (*p* < 0.001).

### 3.3. Comparison of Comorbidities for Survivors and Non-Survivors

From 2016 to 2020, patients with ICD-10 codes indicating a primary diagnosis of SCA had higher rates of in-hospital mortality compared to patients with a primary diagnosis of VT (with secondary SCA diagnosis) or primary diagnosis of VF (*p* < 0.0001). In addition, patients aged 75 and older died at the highest rate compared to other age groups (*p* < 0.0001). The survivors had higher proportion of males (66.3% vs. 57.7%) and patients of white race (68.6% vs. 64.0%) (*p* < 0.0001 for both), as well as higher proportion of hypertension (*p* < 0.0001), congestive heart failure (*p* < 0.0001), ischemic heart disease (*p* < 0.0001), acute coronary syndrome (*p* = 0.000143), non-morbid obesity (*p* < 0.0001), morbid obesity (0.003), and presence of an ICD (*p* < 0.0001). Non-survivors had higher proportion of patients with diabetes, patients insured by Medicare and Medicaid and patients with a median household income within the lowest quartile experienced (*p* < 0.0001 for all) (See Supplementary Table S3). Within the 2020 cohort, a higher proportion of non-survivors had a COVID-19 diagnosis compared to survivors (2.0% vs. 1.1%, *p* = 0.006) (See Supplementary Table S4).

### 3.4. Multivariable Regression Analysis for Associations with Mortality

**Figure 3:**
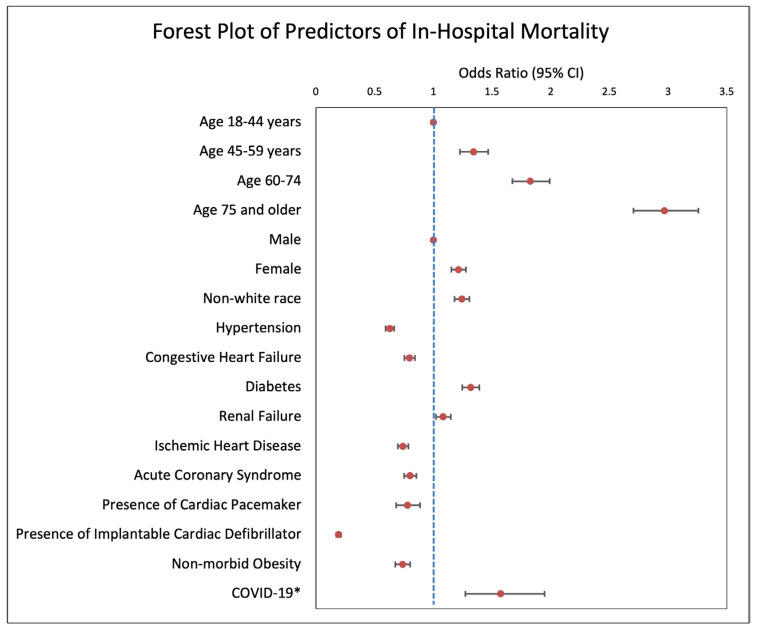
Forest plot presenting adjusted odds ratios for demographic and clinical factors associated with in-hospital mortality among patients hospitalized for sudden cardiac arrest (SCA) from 2016 to 2020. Multivariable logistic regression was used to assess associations. Non-white race, female gender, age > 45, diabetes, renal failure, and COVID-19 * were independently associated with increased mortality. Hypertension, congestive heart failure, ischemic heart disease, acute coronary syndrome, presence of a pacemaker, presence of an ICD, and non-morbid obesity were associated with a lower risk of mortality. * Data is only for the year 2020.

As shown in the forest plot in Figure 3, an analysis of 2020 hospitalizations showed COVID-19 infection to be independently associated with increased mortality among patients with a diagnosis of SCA (OR: 1.57; 95% CI: 1.27–1.95; *p* < 0.001). For the years 2016–2020, hypertension (OR: 0.63; 95% CI: 0.59–0.67; *p* < 0.001) acute coronary syndrome (OR: 0.80; 95% CI: 0.75–0.86; *p* < 0.001), history of congestive heart failure (OR: 0.80; 95% CI: 0.75–0.84; *p* < 0.001), presence of an ICD (OR: 0.19; 95% CI: 0.17–0.21; *p* < 0.001), presence of a pacemaker (OR: 0.78; 95% CI: 0.68–0.89; *p* < 0.001), and non-morbid obesity (OR: 0.74; 95% CI: 0.68–0.80; *p* < 0.001) were found to be associated with lower in-hospital mortality among patients with a diagnosis of SCA (Figure 3). At the same time, age, particularly ages 75 and older (OR: 2.97; CI: 2.70–3.26; *p* < 0.001), non-white race (OR: 1.24; 95% CI: 1.15–1.28; *p* < 0.001), female sex (OR: 1.21; 95% CI: 1.15–1.28; *p* < 0.001), history of renal failure (OR: 1.08; 95% CI: 1.02–1.15; *p* = 0.007), and diabetes (OR: 1.32; 95% CI: 1.25–1.39; *p* < 0.001) were shown to be independently associated with increased in-hospital mortality (Figure 3).

## 4. Discussion

This study compares trends in hospitalizations for sudden cardiac arrest (SCA) in the United States in the years leading up to and during the first year of COVID-19 pandemic. The key findings were as follows: (1) After a gradual increase in the number of hospitalizations for SCA between 2016 and 2019, the number of hospitalizations dropped in 2020. (2) After a gradual decrease in the mortality rates between 2016 and 2019, the data showed a rising trend in mortality rates in 2020. (3) An independent association was documented between COVID-19 and in-hospital mortality among patients hospitalized following SCA. (4) Longer and more costly hospitalizations were documented in patients with a diagnosis of SCA during the COVID-19 year.

The clinical characteristics of the patient population in this study were consistent with prior publications on SCA in regard to the median age and comorbidities including diabetes mellitus (DM), renal failure, congestive heart failure (CHF) and others [15,16,17,18,19]. Various recent publications reported an increase in incidence of out-of-hospital cardiac arrest (OHCA) due to the COVID-19 pandemic [20,21,22]. Uy-Evando et al. identified a reduced proportion of OHCA cases receiving bystander cardiopulmonary resuscitation (CPR) (61% to 51%, respectively; *p* = 0.02), and bystander use of automated external defibrillators (AEDs) (5% to 1%, respectively; *p* = 0.02), as well as prolonged EMS response times (6.6 ± 2.0 min to 7.6 ± 3.0 min, respectively; *p* < 0.001), in 2020 compared to 2019 [23]. This resulted in a lower rate of return of spontaneous circulation (ROSC) in the COVID era, which can explain the decline we observed in SCA hospitalizations, reflecting a reduction in the number of patients who survived long enough to reach the hospital and be hospitalized [23].

Prior studies also report consistently worse survival after OHCA in the years following the onset of the COVID-19 pandemic, with the effects becoming more pronounced during peaks in COVID-19 infections [24,25]. Among other factors, these findings may reflect differences in out-of-hospital management, including variations in the timing and dosing of adrenaline and overall resuscitation efficiency, which were observed to be affected during the COVID-19 pandemic [26]. In addition, Mountantonakis et al. reported increased inpatient mortality for cardiovascular patients during the first wave of the COVID-19 epidemic in New York [27]. This is consistent with our finding of higher in-hospital mortality in 2020 for patients with an SCA diagnosis, compared to the pre-COVID era.

It is important to note that our finding that COVID-19 was documented in only 1.5% of SCA hospitalizations in 2020 is not consistent with prior studies reporting that 5–10% of out-of-hospital cardiac arrests had evidence of acute SARS-CoV-2 infection during the same period [28,29]. This figure likely reflects undercoding of COVID-19 cases, which has been well-documented, especially early in the pandemic when testing and documentation practices were evolving [30]. Therefore, the observed 1.5% almost certainly underestimates the true incidence of COVID-19 among SCA patients during this period.

Interestingly, our data showed a protective effect of hypertension and evidence of coronary heart disease, acute coronary disease or heart failure on survival following SCA hospitalization. This is consistent with some publications demonstrating improved survival post SCA in patients with cardiovascular comorbidities and heart disease [15,31]. This paradoxical finding may be related to better collateral circulation in patients with chronic coronary artery disease and adaptation to ischemic stress in hypertensive patients. Another explanation might lie in higher rate of “shockable rhythm” in patients with prior heart disease, known to be associated with improved outcomes. These patients are also more likely to be receiving beta-blockers, ACE inhibitors or other medications, which may improve post-resuscitation outcomes. Alternatively, these findings may also reflect selection bias, as patients with known heart disease may be more likely to survive to hospitalization, receive more aggressive in-hospital intervention, or be coded differently.

With respect to the effect of COVID-19 on outcomes, a prospective study using patients from UK Biobank identified with COVID-19 infection between March 2020 and November 2020 found that COVID-19 infection, including long-COVID, is associated with increased short- and long-term risks of cardiovascular disease and mortality, consistent with our finding that COVID-19 is independently associated with increased in-hospital mortality [32]. There are various postulated pathophysiological mechanisms suggested to contribute to cardiac arrhythmias associated with COVID-19 infections [33,34]. COVID-19 related cardiomyopathy, has been proposed as a leading cause of OHCA post-COVID-19 infection in an autopsy-based study [35].

Our study has several limitations. First, the data was collected retrospectively from the NIS database that contains discharge-level records and, as such, is susceptible to coding errors, introducing bias. Additionally, data from the post-pandemic era were not available, limiting our ability to evaluate long-term recovery trends in SCA-related hospitalizations. The NIS database also does not include detailed information about patients’ clinical characteristics, including the duration of VT or VF, medication, blood tests, etc. Therefore, we cannot rule out residual confounding within the associations we observed. In addition, SCA may have been under captured due to coding practices, as patients whose primary diagnosis was not SCA, VT, or VF but who had a secondary diagnosis of SCA would not have been included. We chose to focus on patients with SCA as a primary diagnosis (or VT with secondary SCA) to ensure that cases reflected sudden cardiac arrest occurring during the current hospital admission. A final key limitation is the lack of data on the total number of sudden cardiac arrest (SCA) cases occurring in the community, which would serve as the true denominator and provide greater insight into trends at the population level. Unfortunately, our analysis is limited to patients with SCA who survived to hospital admission, as the NIS database does not capture individuals who died before reaching the hospital. We did not have information on total SCA incidence in the population, and our analysis reports hospitalization counts and weighted counts, which cannot be interpreted as population-level rates.

## 5. Conclusions

This study showed that after a steady uptrend in SCA-related admission in the 4 years prior to the COVID pandemic, SCA-related admissions trended downwards in 2020. Among patients admitted with a diagnosis of SCA, no difference in mortality was seen between the pre-COVID era and the COVID outbreak year. The data shows that COVID-19 was independently associated with increased in-hospital mortality in patients hospitalized following SCA. Further research is essential to investigate the underlying mechanisms that may explain this association.

## Figures and Tables

**Table 1:** Baseline characteristics of patients hospitalized with SCA before (2016–2019) and during (2020) the COVID-19 era.

		Year			
		2016–2019	2020	Total	*p* Value ^1^
Patients, n	Unweighted	6226 (avg)	5717	30,620	0.066
	Weighted	31,129	28,585	153,100	
Primary diagnosis, %	VT	9.7%	10.7%	9.9%	0.041 *
	VF	42.9%	43.7%	43.0%	0.26
	SCA	47.4%	45.6%	47.0%	0.019 *
Age group, %	18–44	10.7%	11.4%	10.8%	0.007 *
	45–59	23.9%	23.7%	23.8%	
	60–74	38.3%	39.7%	38.6%	
	75 and older	27.2%	25.2%	26.8%	
Gender, %	Male	62.8%	62.3%	62.7%	0.40
	Female	37.2%	37.7%	37.3%	
Race, %	White	66.7%	66.4%	66.6%	0.12
	Black	19.1%	19.1%	19.1%	
	Hispanic	7.9%	7.4%	7.8%	
	Asian/Pacific Islander	2.7%	2.6%	2.6%	
	Native American	0.6%	0.8%	0.6%	
	Other	3.2%	3.7%	3.2%	
Comorbidities, %	Hypertension	68.3%	68.4%	68.4%	0.98
	Congestive Heart Failure	28.8%	30.3%	29.1%	0.029 *
	Diabetes	34.1%	32.6%	33.8%	0.026 *
	Renal Failure	28.6%	28.8%	28.7%	0.84
	Ischemic Heart Disease	23.7%	22.0%	23.3%	0.009 *
	Acute Coronary Syndrome	14.7%	18.5%	15.4%	<0.001 *
	Peripheral Vascular Disease	7.1%	6.3%	6.9%	0.070
	Cardiac Pacemaker	3.5%	3.0%	3.4%	0.047 *
	Implantable Cardiac Defibrillator	11.7%	11.0%	11.6%	0.16
	Non-morbid obesity	8.9%	9.9%	9.1%	0.012 *
	Morbid obesity	7.9%	8.4%	8.0%	0.23
Deyo-CCI, %	0	15.7%	15.5%	15.6%	0.22
	1	21.7%	20.7%	21.5%	
	2 or higher	62.7%	63.8%	62.9%	
Primary expected payer, %	Medicare	55.5%	52.5%	54.9%	0.0016 *
	Medicaid	13.2%	14.3%	13.4%	
	Private	23.7%	24.5%	23.8%	
	Self-pay	4.4%	5.1%	4.6%	
	No Charge	0.2%	0.3%	0.3%	
	Other	3.0%	3.3%	3.0%	
Median household income, %	0 to 25th percentile	30.7%	30.7%	30.7%	0.93
	26th to 50th percentile	26.1%	26.5%	26.2%	
	51st to 75th percentile	23.5%	23.4%	23.4%	
Hospital status, %	Rural	5.3%	4.6%	5.1%	<0.001 *
	Urban nonteaching	20.7%	17.5%	20.0%	
	Urban teaching	74.1%	77.9%	74.9%	
Hospital region, %	Northeast	16.4%	15.0%	16.2%	0.080
	Midwest	22.6%	22.8%	22.6%	
	South	41.5%	42.6%	41.7%	
	West	19.5%	19.6%	19.5%	
Hospital bedsize, %	Small	16.3%	18.9%	16.8%	<0.001 *
	Medium	29.4%	28.7%	29.3%	
	Large	54.3%	52.4%	53.9%	

^1^ *p*-values were generated using the chi-square test and refer to changes in frequency before and after the COVID year (2020). Values marked with * are statistically significant (*p* < 0.05).

**Table 2:** Outcomes of patients hospitalized with SCA in the U.S. before (2016–2019) and during (2020) the COVID-19 era.

		Year			
		2016–2019	2020	Total	*p* Value ^1^
In-hospital interventions, %	Coronary catheterization	25.2%	23.9%	25.0%	0.04 *
	Pacemaker implantation	1.1%	0.9%	1.0%	0.38
	ICD insertion	15.8%	16.9%	16.0%	0.02 *
Mortality (total and by diagnosis), %	VT	28.8%	28.7%	28.7%	0.99
	VF	23.8%	24.5%	23.9%	0.44
	SCA	73.2%	73.5%	73.2%	0.77
	Total	47.7%	47.3%	47.6%	0.66
Disposition of patient, %	Routine	47.9%	48.5%	48.0%	<0.001 *
	Transfer to Short-term Hospital	12.1%	10.4%	11.8%	
	Transfer other	26.1%	24.6%	25.9%	
	Home Health Care	12.7%	15.3%	13.2%	
	Against Medical Advice	1.0%	1.0%	1.0%	
	Discharge alive, unknown destination	0.2%	0.2%	0.2%	
Length of stay, mean days		5.71	6.08	5.78	0.008 *
Total charges, mean dollars		122,659	144,786	127,096	<0.001 *

^1^ *p*-values were generated using the chi-square test and refer to changes in frequency before and after the COVID year (2020). Values marked with * are statistically significant (*p* < 0.05).

## Data Availability

The original data presented in this study are openly available in the HCUP Nationwide Inpatient Sample (NIS) database at https://hcup-us.ahrq.gov/db/nation/nis/NIS_Introduction_2020.jsp (accessed on 23 December 2022).

## Supplementary Materials

The following supporting information can be downloaded at https://www.mdpi.com/article/10.3390/jcm14217517/s1, Table S1: Trends in Baseline Characteristics of Patients Hospitalized with Atrial Fibrillation in the U.S. Between 2016 and 2020; Table S2: Trends in Outcomes of Patients Hospitalized with SCA in the U.S. Between 2016 and 2020; Table S3: Comparison of Patients with SCA Diagnosis by In-Hospital Mortality, 2016–2020; Table S4: Comparison of Patients with SCA Diagnosis by In-Hospital Mortality in 2020 only.

## Abbreviations

The following abbreviations are used in this manuscript:

SCA	sudden cardiac arrest
OHCA	out-of-hospital cardiac arrest
VF	ventricular fibrillation
VT	ventricular tachycardia
ICD	implantable cardioverter-defibrillator
CHF	congestive heart failure
CPR	cardiopulmonary resuscitation
AED	automated external defibrillator
ROSC	return of spontaneous circulation
ACE	angiotensin-converting enzyme

## Appendix A

**Table A1:** ICD-10 CM diagnosis and procedure codes [36].

Condition	ICD-10 CM Codes
SCD	I46.2, I46.8, I46.9
VT	I49.01, I49.02
VF	I47.20, I47.21, I47.29
Hypertension	I10, I11.0, I11.9, I12.0, I12.9, I13.0, I13.10, I13.11, I13.2 I15.0, I15.1, I15.2, I15.8, I15.9, I16.0, I16.1, I16.9
Congestive Heart Failure	I50.1, I50.20, I50.22, I50.30, I50.32, I50.40, I50.42, I50.810, I50.812, I50.814, I50.82, I50.83, I50.84, I50.89, I50.9
History of Ischemic Heart Disease	I25.2, Z95.5, Z95.1, Z95.8
Diabetes Mellitus	E08.42, E08.649, E08.65, E08.9, E09.22, E09.649, E09.65, E09.8, E09.9, E10.0x, E10.1x, E10.6x, E10.8x, E10.9x, E11.0x, E11.1x, E11.6x, E11.8x, E11.9x, E13.0x, E13.1x, E13.6x, E13.8x, E13.9x
Renal Failure	I12.0, I12.9, I13.0, I13.10, I13.11, I13.2, N17.0, N17.1, N17.2, N17.8, N17.9, N18.1, N18.2, N18.3, N18.30, N18.31, N18.32, N18.4, N18.5, N18.6, N18.9, N19, Z49.01, Z49.02, Z49.31, Z49.32, Z94.0, Z99.2
COVID-19	U07.1, J12.82
Acute Coronary Syndrome	I20.0, I20.1, I21.01, I21.02, I21.09, I21.11, I21.19 I21.21, I21.29, I21.3, I21.4, I21.9, I21.A1, I21.A9, I21.B, I22.0, I22.1, I22.2, I22.8, I22.9, I23.0, I24.0, I24.8, I24.9
Peripheral Vascular Disease	I70.0, I70.1, I70.2x, I70.3x, I70.4x, I70.5x, I70.6x, I70.7x, I70.8x, I70.9x, I73.1, I73.8, I73.9, I771, I79.0, I79.2, K55.1, K55.8, K55.9, Z95.8, Z95.9
Cardiac Pacemaker	Z95.0
Implantable Cardiac Defibrillator	Z95.810
Pacemaker implantation	0JH634Z, 0JH635Z, 0JH636Z, 0JH637Z, 0JH63MZ, 0JH604Z, 0JH605Z, 0JH606Z, 0JH607Z
ICD insertion	0JH608Z, 0JH609Z, 0JH638Z, 0JH639Z, 0JH808Z, 0JH809Z, 0JH838Z, 0JH839Z, 0JH60FZ, 0JH63FZ, 02H43KZ, 02H60KZ, 02H63KZ, 02H64KZ, 02H70KZ, 02H73KZ, 02H74KZ, 02HK0KZ, 02HK3KZ, 02HK4KZ, 02HL0KZ, 02HL3KZ, 02HL4KZ
Catheter ablation	025K0ZZ, 025K3ZZ, 025K4ZZ, 025L0ZZ, 025L3ZZ, 025L4ZZ, 025M0ZZ, 025M3ZZ, 025M4ZZ, 025D0ZZ, 025D3ZZ, 025D4ZZ
Coronary catheterization	4A023N6, 4A023N7, 4A023N8

**Table A2:** ICD-10 CM codes for Deyo-CCI score [15].

Condition	ICD-10 CM Codes for Deyo-CCI Score	Score
Myocardial infarction	I21.x, I22.x, I25.2	1
Congestive heart failure	I09.9, I11.0, I13.0, I13.2, I25.5, I42.0, I42.5–I42.9, I43.x, I50.x, P29.0	1
Peripheral vascular disease	I70.x, I71.x, I73.1, I73.8, I73.9, I77.1, I79.0, I79.2, K55.1, K55.8, K55.9, Z95.8, Z95.9	1
Cerebrovascular disease	G45.x, G46.x, H34.0, I60.x–I69.x	1
Dementia	F00.x–F03.x, F05.1, G30.x, G31.1	1
Chronic pulmonary disease	I27.8, I27.9, J40.x–J47.x, J60.x–J67.x, J68.4, J70.1, J70.3	1
Rheumatologic disease	M05.x, M06.x, M31.5, M32.x–M34.x, M35.1, M35.3, M36.0	1
Peptic ulcer disease	K25.x–K28.x	1
Mild liver disease	B18.x, K70.0–K70.3, K70.9, K71.3–K71.5, K71.7, K73.x, K74.x, K76.0, K76.2–K76.4, K76.8, K76.9, Z94.4	1
Diabetes	E10.0, E10.l, E10.6, E10.8, E10.9, E11.0, E11.1, E11.6, E11.8, E11.9, E12.0, E12.1, E12.6, E12.8, E12.9, E13.0, E13.1, E13.6, E13.8, E13.9, E14.0, E14.1, E14.6, E14.8, E14.9	1
Diabetes with chronic complications	E10.2–E10.5, E10.7, E11.2–E11.5, E11.7, E12.2–E12.5, E12.7, E13.2–E13.5, E13.7, E14.2–E14.5, E14.7	2
Hemiplegia or paraplegia	G04.1, G11.4, G80.1, G80.2, G81.x, G82.x, G83.0–G83.4, G83.9	2
Renal disease	I12.0, I13.1, N03.2–N03.7, N05.2–N05.7, N18.x, N19.x, N25.0, Z49.0–Z49.2, Z94.0, Z99.2	2
Any malignancy including leukemia and lymphoma	C00.x–C26.x, C30.x–C34.x, C37.x–C41.x, C43.x, C45.x–C58.x, C60.x–C76.x, C81.x–C85.x, C88.x, C90.x–C97.x	2
Moderate or severe liver disease	I85.0, I85.9, I86.4, I98.2, K70.4, K71.1, K72.1, K72.9, K76.5, K76.6, K76.7	3
Metastatic solid tumor	C77.x–C80.x	6
Acquired immunodeficiency syndrome (AIDS)	B20.x–B22.x, B24.x	6
