# Supplementary material for: Nationwide Trends in Hospitalizations for Sudden Cardiac Arrest Before and During the COVID Outbreak
Source: J Clin Med. 2025 Oct 23;14(21):7517. doi: 10.3390/jcm14217517 (PMC12607978; doi:10.3390/jcm14217517)
Supplement: Supplementary file 1 [file jcm-14-07517-s001.zip › Supplementary Table S1.pdf]

Supplementary Table S1. Trends in Baseline Characteristics of Patients Hospitalized with Atrial Fibrillation in the U.S. Between 2016 and 2020

		Year						
		**2016**	**2017**	**2018**	**2019**	**2020**	**Total**	**P-value**
**Patients, n**	Unweighted	5704	6272	6473	6454	5717	30620	0.89
	Weighted	28520	31360	32365	32270	28585	153100	0.89
**Primary diagnosis, %**	VT	8.9%	9.3%	10.2%	10.6%	10.7%	9.9%	0.000095
	VF	42.9%	41.7%	42.0%	45.0%	43.7%	43.0%	0.012
	SCA	48.2%	49.0%	47.8%	44.5%	45.6%	47.0%	0.0000014
**Age group, %**	18-44	10.8%	10.4%	10.2%	11.1%	11.4%	10.8%	0.51
	45-59	24.2%	24.0%	23.8%	23.4%	23.7%	23.8%	
	60-74	38.4%	38.3%	38.4%	38.0%	39.7%	38.6%	
	75 and older	26.6%	27.2%	27.5%	27.5%	25.2%	26.8%	

**Gender, %**	Male	61.7%	62.6%	63.1%	63.9%	62.3%	62.7%	0.20
	Female	38.3%	37.4%	36.9%	36.1%	37.7%	37.3%	
**Race, %**	White	68.0%	66.0%	66.4%	66.2%	66.4%	66.6%	0.036
	Black	18.4%	19.7%	18.7%	19.5%	19.1%	19.1%	
	Hispanic	7.3%	7.8%	8.4%	8.1%	7.4%	7.8%	
	Asian/Pacific Islander	2.8%	2.6%	2.6%	2.7%	2.6%	2.6%	
	Native American	0.4%	0.7%	0.6%	0.6%	0.8%	0.6%	
	Other	3.1%	3.2%	3.4%	2.9%	3.7%	3.2%	
**Comorbidities, %**	Hypertension	66.6%	67.7%	69.0%	69.9%	68.4%	68.4%	0.0022
	Congestive Heart Failure	25.8%	28.5%	29.9%	30.9%	30.3%	29.1%	<0.001
	Diabetes	33.0%	34.2%	34.7%	34.5%	32.6%	33.8%	0.79
	Renal Failure	26.9%	28.6%	29.1%	29.9%	28.8%	28.7%	0.0059
	Ischemic Heart Disease	24.7%	23.3%	23.9%	22.7%	22.0%	23.3%	0.00070

	Acute Coronary Syndrome	12.8 %	13.6 %	15.3 %	17.0 %	18.5 %	15.4%	<0.001
	Peripheral Vascular Disease	8.5%	6.9%	6.4%	6.4%	6.3%	6.9%	<0.001
	Cardiac Pacemaker	3.6%	3.7%	3.3%	3.4%	3.0%	3.4%	0.040
	Implantable Cardiac Defibrillator	11.8 %	11.6 %	11.7 %	11.7 %	11.0 %	11.6%	0.28
**Deyo-CCI, %**	0	16.9 %	16.2 %	15.6 %	14.1 %	15.5 %	15.6%	<0.001
	1	22.1 %	22.2 %	21.3 %	21.0 %	20.7 %	21.5%	
	2 or higher	61.0 %	61.5 %	63.2 %	64.9 %	63.8 %	62.9%	
**Primary expected payer, %**	Medicare	56.2 %	55.1 %	55.5 %	55.3 %	52.5 %	54.9%	0.039
	Medicaid	13.1 %	13.5 %	13.0 %	13.0 %	14.3 %	13.4%	
	Private	23.6 %	23.9 %	23.8 %	23.4 %	24.5 %	23.8%	
	Self-pay	4.0%	4.2%	4.6%	5.1%	5.1%	4.6%	
	No Charge	0.2%	0.3%	0.2%	0.2%	0.3%	0.3%	
	Other	3.0%	3.0%	2.9%	3.0%	3.3%	3.0%	

**Median household income, %**	0 to 25th percentile	31.3%	30.7%	29.5%	31.2%	30.7%	30.7%	0.61
	26th to 50th percentile	25.4%	26.7%	27.1%	25.2%	26.5%	26.2%	
	51st to 75th percentile	23.7%	23.0%	23.5%	23.7%	23.4%	23.4%	
	76th to 100th percentile	19.6%	19.6%	19.9%	19.9%	19.5%	19.7%	
**Hospital status, %**	Rural	5.5%	5.5%	5.1%	5.0%	4.6%	5.1%	<0.001
	Urban nonteaching	24.5%	21.6%	19.7%	16.9%	17.5%	20.0%	
	Urban teaching	70.0%	72.9%	75.2%	78.1%	77.9%	74.9%	
**Hospital region, %**	Northeast	16.5%	16.8%	16.9%	15.5%	15.0%	16.2%	0.33
	Midwest	23.0%	22.1%	22.2%	23.0%	22.8%	22.6%	
	South	40.9%	41.0%	41.5%	42.4%	42.6%	41.7%	
	West	19.5%	20.1%	19.4%	19.1%	19.6%	19.5%	
**Hospital bedsize, %**	Small	14.2%	16.1%	17.0%	17.9%	18.9%	16.8%	<0.001

	Medium	28.6%	29.9%	29.6%	29.5%	28.7%	29.3%
	Large	57.2%	54.0%	53.4%	52.6%	52.4%	53.9%

P-values were generated using the chi-square test.
