# Supplementary material for: Nationwide Trends in Hospitalizations for Sudden Cardiac Arrest Before and During the COVID Outbreak
Source: J Clin Med. 2025 Oct 23;14(21):7517. doi: 10.3390/jcm14217517 (PMC12607978; doi:10.3390/jcm14217517)
Supplement: Supplementary file 1 [file jcm-14-07517-s001.zip › Supplementary Table S2.pdf]

Supplementary Table S2. Trends in Outcomes of Patients Hospitalized with SCA in the U.S. Between 2016 and 2020

		Year						
		**2016**	**2017**	**2018**	**2019**	**2020**	**Total**	**P-value**
**Outcomes, %**	Pacemaker implantation	1.3%	1.1%	1.1%	0.9%	0.9%	1.0%	0.025
	ICD insertion	15.9%	15.3%	15.9%	16.3%	16.9%	16.0%	0.72
	Catheter ablation	0.1%	0.0%	0.0%	0.0%	0.0%	0.1%	0.13
	Coronary catheterization	25.3%	25.3%	25.2%	25.2%	23.9%	25.0%	0.058
	Mortality	48.3%	48.8%	47.7%	45.8%	47.3%	47.6%	0.012
**Mortality by Diagnosis, %**	VT	28.5%	30.1%	27.3%	29.2%	28.7%	28.7%	0.93
	VF	22.9%	24.8%	23.4%	23.9%	24.5%	23.9%	0.43
	SCA	74.5%	72.8%	73.4%	72.0%	73.5%	73.2%	0.26
**Disposition of patient, %**	Routine	49.0%	48.3%	47.5%	46.8%	48.5%	48.0%	0.053
	Transfer to Short-term Hospital	12.2%	12.3%	11.9%	12.1%	10.4%	11.8%	
	Transfer other	25.6%	26.7%	25.7%	26.6%	24.6%	25.9%	
	Home Health Care	12.2%	11.6%	13.4%	13.4%	15.3%	13.2%	

	Against Medical Advice	0.8%	1.1%	1.2%	1.0%	1.0%	1.0%	
	Discharge alive, unknown destination	0.2%	0.0%	0.3%	0.1%	0.2%	0.2%	
**Length of stay, mean days**		5.71	5.69	5.54	5.91	6.08	5.78	0.012
**Total charges, mean dollars**		111921	119437	122924	136353	144786	127096	<0.001
P-values were generated using the chi-square test.								
