# Supplementary material for: Nationwide Trends in Hospitalizations for Sudden Cardiac Arrest Before and During the COVID Outbreak
Source: J Clin Med. 2025 Oct 23;14(21):7517. doi: 10.3390/jcm14217517 (PMC12607978; doi:10.3390/jcm14217517)
Supplement: Supplementary file 1 [file jcm-14-07517-s001.zip › Supplementary Table S3.pdf]

Supplementary Table S3. Comparison of Patients with SCA Diagnosis by In-Hospital Mortality, 2016-2020

		**Died during hospitalization**			
		**Survivors**	**Non-survivors**	**Total**	**P-value**
**Patients, n**	Unweighted	16054	14566	30620	
	Weighted	80270	72830	153100	
**Primary diagnosis, %**	VT	13.5%	6.0%	9.9%	<0.0001
	VF	62.5%	21.6%	43.0%	<0.0001
	SCA	24.0%	72.4%	47.0%	<0.0001
**Age group**	18-44	12.5%	8.9%	10.8%	<0.0001
	45-59	26.6%	20.7%	23.8%	
	60-74	39.3%	37.7%	38.6%	
	75 and older	21.5%	32.7%	26.8%	
**Gender, %**	Male	66.3%	58.8%	62.7%	<0.0001
	Female	33.7%	41.2%	37.3%	
**Race, %**	White	68.1%	64.9%	66.6%	<0.0001
	Black	18.8%	19.4%	19.1%	
	Hispanic	7.2%	8.5%	7.8%	
	Asian/Pacific Islander	2.5%	2.8%	2.6%	
	Native American	0.5%	0.7%	0.6%	
	Other	2.9%	3.6%	3.2%	
**Comorbidities, %**	Hypertension	72.1%	64.2%	68.4%	<0.0001

	Congestive Heart Failure	33.2%	24.7%	29.1%	<0.0001
	Diabetes mellitus	32.2%	35.6%	33.8%	<0.0001
	Renal Failure	28.5%	29.0%	28.7%	0.36
	Ischemic Heart Disease	27.4%	18.8%	23.3%	<0.0001
	Acute Coronary Syndrome	16.2%	14.6%	15.4%	0.00014
	Peripheral Vascular Disease	7.1%	6.7%	6.9%	0.19
	Cardiac Pacemaker	3.5%	3.3%	3.4%	0.61
	Implantable Cardiac Defibrillator	18.7%	3.7%	11.6%	<0.0001
	COVID-19	0.2%	0.4%	0.3%	0.0069
**Deyo-CCI, %**	0	13.1%	18.4%	15.6%	<0.0001
	1	21.8%	21.0%	21.5%	
	2 or higher	65.1%	60.5%	62.9%	
**Obesity, %**	Non-morbid	10.8%	7.2%	9.1%	<0.0001
	Morbid	8.4%	7.5%	8.0%	0.0029
**Primary expected payer, %**	Medicare	50.2%	60.1%	54.9%	<0.0001
	Medicaid	13.1%	13.6%	13.4%	
	Private	29.9%	17.2%	23.8%	
	Self-pay	3.3%	6.0%	4.6%	
	No Charge	0.3%	0.3%	0.3%	
	Other	3.2%	2.9%	3.0%	
**Median household income, %**	0 to 25th percentile	28.5%	33.1%	30.7%	<0.0001
	26th to 50th percentile	26.2%	26.2%	26.2%	

	51st to 75th percentile	24.1%	22.7%	23.4%	
	76th to 100th percentile	21.2%	18.0%	19.7%	
**Hospital Status, %**	Rural	4.8%	5.5%	5.1%	0.032
	Urban nonteaching	20.1%	19.8%	20.0%	
	Urban teaching	75.1%	74.7%	74.9%	
**Hospital Region, %**	Northeast	15.7%	16.7%	16.2%	<0.0001
	Midwest	23.2%	21.9%	22.6%	
	South	41.1%	42.3%	41.7%	
	West	20.0%	19.1%	19.5%	
**Hospital Bedsize, %**	Small	16.3%	17.5%	16.8%	<0.0001
	Medium	29.1%	29.5%	29.3%	
	Large	54.6%	53.1%	53.9%	
P-values were generated using the chi-square test.					
