# Supplementary material for: Nationwide Trends in Hospitalizations for Sudden Cardiac Arrest Before and During the COVID Outbreak
Source: J Clin Med. 2025 Oct 23;14(21):7517. doi: 10.3390/jcm14217517 (PMC12607978; doi:10.3390/jcm14217517)
Supplement: Supplementary file 1 [file jcm-14-07517-s001.zip › Supplementary Table S4.pdf]

Supplementary Table S4. Comparison of Patients with SCA Diagnosis by In-Hospital Mortality in 2020 only

		Died during hospitalization			
		Survivors	Non-survivors	Total	P-value
**Primary diagnosis, %**	VT	14.4%	6.5%	10.7%	<0.001
	VF	62.6%	22.7%	43.7%	<0.001
	SCA	23.0%	70.9%	45.6%	<0.001
**Age group, %**	18-44	12.7%	10.0%	11.4%	<0.001
	45-59	26.3%	20.8%	23.7%	
	60-74	40.7%	38.6%	39.7%	
	75 and older	20.3%	30.6%	25.2%	
**Gender, %**	Male	66.3%	57.7%	62.3%	<0.001
	Female	33.7%	42.3%	37.7%	
**Race, %**	White	68.6%	64.0%	66.4%	0.0066
	Black	18.3%	20.1%	19.1%	
	Hispanic	6.8%	8.1%	7.4%	
	Asian/Pacific Islander	2.4%	2.8%	2.6%	
	Native American	0.6%	1.0%	0.8%	
	Other	3.4%	4.0%	3.7%	
**Comorbidities, %**	Hypertension	72.7%	63.6%	68.4%	<0.001
	Congestive Heart Failure	34.2%	26.0%	30.3%	<0.001

	Diabetes	30.4%	35.0%	32.6%	<0.001
	Renal Failure	29.1%	28.4%	28.8%	0.58
	Ischemic Heart Disease	25.7%	17.9%	22.0%	<0.001
	Acute Coronary Syndrome	20.3%	16.4%	18.5%	<0.001
	Peripheral Vascular Disease	6.9%	5.8%	6.3%	0.099
	Cardiac Pacemaker	3.1%	2.8%	3.0%	0.65
	Implantable Cardiac Defibrillator	18.0%	3.3%	11.0%	<0.001
	COVID-19	1.1%	2.0%	1.5%	0.0056
**Deyo-CCI, %**	0	12.3%	19.1%	15.5%	<0.001
	1	21.4%	19.8%	20.7%	
	2 or higher	66.3%	61.1%	63.8%	
**Obesity, %**	Non-morbid	12.5%	7.1%	10.0%	<0.001
	Morbid	8.8%	7.9%	8.4%	
**Primary expected payer, %**	Medicare	48.3%	57.2%	52.5%	<0.001
	Medicaid	14.3%	14.2%	14.3%	
	Private	30.1%	18.3%	24.5%	
	Self-pay	3.6%	6.8%	5.1%	
	No Charge	0.3%	0.4%	0.3%	
	Other	3.4%	3.1%	3.3%	
**Median household income, %**	0 to 25th percentile	28.9%	32.6%	30.7%	0.0028
	26th to 50th percentile	26.4%	26.5%	26.5%	
	51st to 75th percentile	23.6%	23.1%	23.4%	

	76th to 100th percentile	21.0%	17.7%	19.5%	
**Hospital status, %**	Rural	4.4%	4.9%	4.6%	0.69
	Urban nonteaching	17.5%	17.5%	17.5%	
	Urban teaching	78.1%	77.6%	77.9%	
**Hospital region, %**	Northeast	14.8%	15.3%	15.0%	0.27
	Midwest	23.3%	22.2%	22.8%	
	South	41.6%	43.7%	42.6%	
	West	20.3%	18.8%	19.6%	
**Hospital Bedsize, %**	Small	18.7%	19.0%	18.9%	0.84
	Medium	29.0%	28.3%	28.7%	
	Large	52.2%	52.7%	52.4%	
P-values were generated using the chi-square test.					
